# Borrowing sunlight: Photosynthetic bioengineering as an emerging therapeutic paradigm

**DOI:** 10.1002/ctm2.70816

**Published:** 2026-08-31

**Authors:** Fangyu Zhou, Nengyi Ni, Kuoran Xing, Xiao Sun

**Affiliations:** ^1^ Shandong Cancer Hospital and Institute Shandong First Medical University and Shandong Academy of Medical Sciences Jinan China; ^2^ Department of Chemical and Biomolecular Engineering College of Design and Engineering National University of Singapore Singapore Singapore

Light is one of the most abundant and continuously available energy sources on Earth, but unlike plant cells, mammalian cells lack the ability to directly convert the abundant light energy into biochemical energy. In recent years, a series of studies have begun to attempt to cross this bioenergy boundary, introducing plant‐derived thylakoids and their light‐response functions into inflamed joints, damaged kidneys, infarcted myocardium and, most recently, corneal tissue (Figure 1A),^1^, ^2^, ^3^, ^4^, ^5^, ^6^, ^7^ to explore a common question: Can the mechanism of photosynthesis be used to provide controllable local energy and reducing power to mammalian tissues? Individually, these works are proof‐of‐concept for different organs; collectively, they are outlining a new therapeutic paradigm—photosynthetic medicine. This emerging concept of ‘photosynthetic medicine’ encompasses therapeutic strategies that convert exogenous light energy into locally usable chemical energy by transplanting or reconstructing photosynthetic machinery in mammalian systems.

**FIGURE 1:**
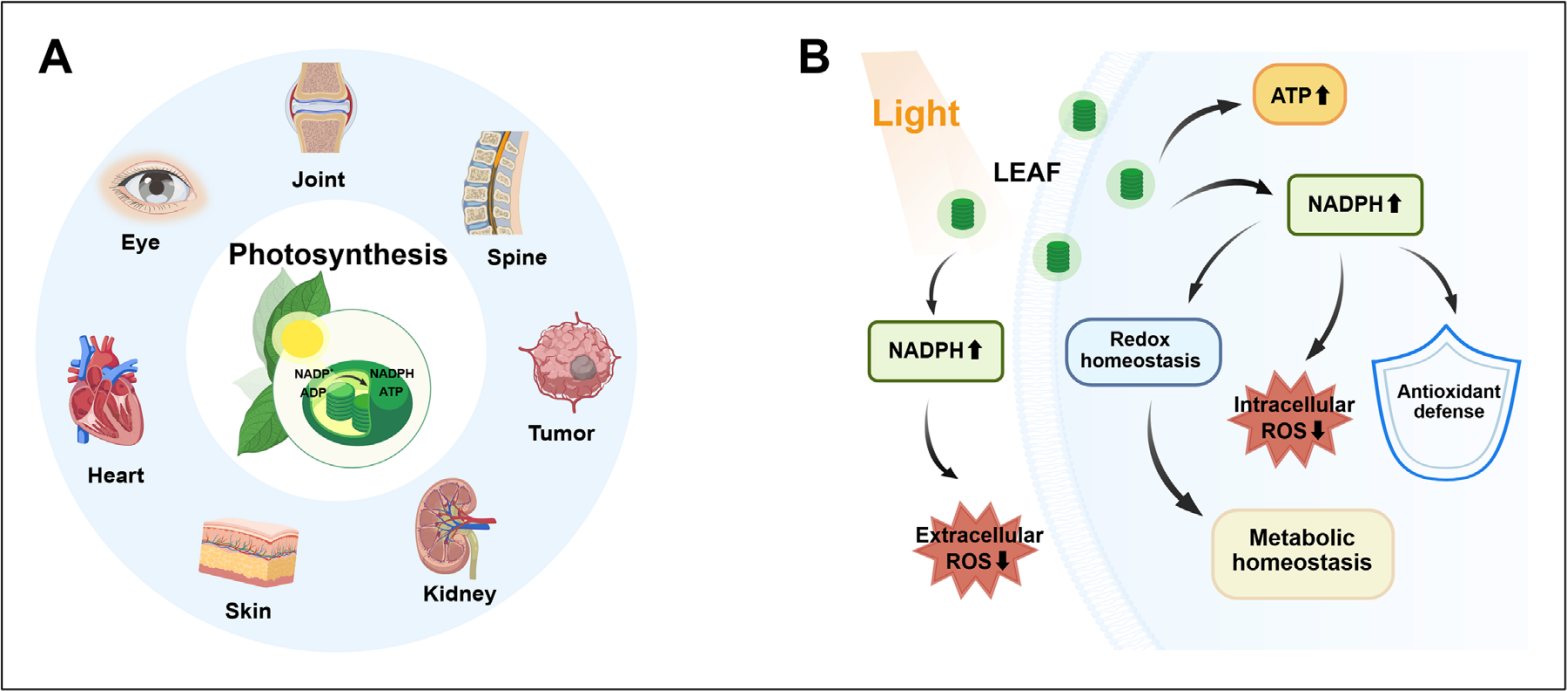
Emerging therapeutic applications and bioenergetic mechanism of photosynthetic systems in mammalian tissues. (A) Representative applications of plant‐derived photosynthetic systems across different tissues and disease settings. (B) Schematic illustration of the bioenergetic actions of the LEAF (light‐reaction enriched thylakoid NADPH‐foundry) nanosystem in corneal tissue. The figure was created with BioGDP.com^9^

## 1 THE BIOENERGETIC PROMISE AND LIMITATIONS OF THYLAKOID SYSTEMS

These studies share a common thread: the use of thylakoids derived from chloroplasts and, more importantly, the validation of a shared scientific hypothesis. The light‐dependent reactions of photosynthesis can be decoupled to some extent from other chloroplast functions, temporarily maintaining their energy conversion capabilities in mammalian systems. Its potential application value lies in the fact that light may serve as a continuous external energy input, converted into locally available reducing power and chemical energy. For example, NADPH (reduced nicotinamide adenine dinucleotide phosphate) is a key coenzyme for maintaining cellular redox homeostasis and antioxidant defence;^8^ its depletion is widely involved in inflammation and ischemic injury. Continuous light‐driven NADPH replenishment may therefore offer a distinct alternative to stoichiometric antioxidant or anti‐inflammatory therapies.

However, this strategy still faces key engineering and translational hurdles. The separated thylakoid structure is relatively fragile, and its photoreaction products may be consumed by residual chloroplast components, thus limiting the net output to the host cell.

## 2 LEAF: FROM PROOF OF CONCEPT TO TRANSLATIONAL APPLICATION

The recently developed LEAF (light‐reaction enriched thylakoid NADPH‐foundry) has further promoted this strategy from proof of concept to engineering and translational application.^1^ By selectively removing components in chloroplasts that consume NADPH while retaining thylakoid grana and their photoreaction function, LEAF improves the net output of NADPH, compared to untreated thylakoids. After topical administration, LEAF can be taken up by corneal cells and maintain photosynthetic electron transport activity, providing photogenerated NADPH and ATP (adenosine triphosphate) in the cell; at the same time, NADPH released into the local environment can enhance antioxidant defence and reduce reactive oxygen species levels (Figure 1B). Thus, LEAF advances the thylakoid light response from simple ‘functional preservation’ to energy and redox support that can be utilised by host tissues.

More importantly, this study establishes a more clinically relevant validation chain. LEAF not only demonstrates therapeutic effects in animal models but also increases NADPH levels in tear samples from human dry eye patients and is further compared with clinically used cyclosporine eye drops. Combined with its topical mode of administration, short‐term safety and clear improvement in corneal function, this chain of evidence brings photosynthetic bioengineering closer from mechanistic proof‐of‐concept to a transferable therapeutic strategy.

## 3 FROM OPHTHALMIC PROOF‐OF‐CONCEPT TO THERAPEUTIC PLATFORM

Conceptually, LEAF positions photosynthetic machinery as a transferable orthogonal bioenergy module rather than simply a therapeutic material. By converting external light into bioavailable reducing power and chemical energy, such systems may expand the metabolic capacity of mammalian tissues.

From the application side, the ocular surface represents a particularly realistic entry point, combining the physiological light exposure with convenient local delivery, while deeper tissues are limited by light penetration.

From a translational point of view, photosynthetic therapy demands a different dosage and evaluation framework than traditional drugs. Its bioactivity will require dosage metrics that integrate the amount of material with the intensity of light and duration of exposure. Long‐term safety, immunogenicity, degradation and batch consistency will be important for reproducible clinical use.

A logical next step for LEAF is to look at tissues that share the key advantages of the ocular surface: ready access to light and feasibility of local administration. Skin and exposed wounds are particularly good candidates. Light‐driven bioenergetic support can work outside the eye, as demonstrated by the therapeutic potential of photosynthetic thylakoid‐based systems for chronic wound healing.^6^ But it is not known if the increased net NADPH output of LEAF provides a measurable advantage over standard thylakoid preparations. The incorporation of LEAF into hydrogels, wound dressings or other locally retained formulations could allow for sustained illumination and ongoing provision of reducing power in chronic wounds, burns or other superficial injuries characterised by oxidative stress and impaired tissue repair. Such studies should involve direct comparisons between LEAF and conventional thylakoids at matched light doses and quantify NADPH availability, redox recovery, cellular regeneration and tissue healing. More broadly, light accessibility, together with local delivery feasibility and metabolic demand, may provide a practical framework for selecting future indications for photosynthetic medicine.

## 4 CONCLUSION

Introducing light‐dependent responses into mammalian ocular tissues should not be viewed merely as a proof‐of‐concept for a single disease but rather as an important example of a broader therapeutic concept: non‐mammalian energy conversion mechanisms can be engineered to provide controllable metabolic support for mammalian tissues. LEAF further combines this concept with local delivery, physiological light exposure and clinically relevant validation, propelling photosynthetic bioengineering towards translational applications. Therefore, the key issue in the next stage is no longer whether the photosynthetic mechanism can function in mammalian tissues but how to make it sufficiently controllable, stable and safe so as to develop it into a reproducible and standardised treatment strategy.

## CONFLICT OF INTEREST STATEMENT

The authors declare no conflicts of interest.

## FUNDING INFORMATION

This study was supported by the Natural Science Foundation of Shandong Province (No. ZR2025LZL006).
